# Centrally Acting Angiotensin-Converting Enzyme Inhibitor Suppresses Type I Interferon Responses and Decreases Inflammation in the Periphery and the CNS in Lupus-Prone Mice

**DOI:** 10.3389/fimmu.2020.573677

**Published:** 2020-09-15

**Authors:** Cassandra Nocito, Cody Lubinsky, Michelle Hand, Sabeeya Khan, Tulsi Patel, Alecia Seliga, Malika Winfield, Viviana Zuluaga-Ramirez, Nicole Fernandes, Xiangdang Shi, Ellen M. Unterwald, Yuri Persidsky, Uma Sriram

**Affiliations:** ^1^Department of Pathology and Laboratory Medicine, Temple University, Philadelphia, PA, United States; ^2^Center for Substance Abuse Research, Lewis Katz School of Medicine, Temple University, Philadelphia, PA, United States

**Keywords:** type I interferon, angiotensin-converting enzyme inhibitor, captopril, immune complex, neuroinflammation, serotonin, depression, lupus-prone mice

## Abstract

Systemic lupus erythematosus (SLE) is an autoimmune disease characterized by multi-organ damage. Neuropsychiatric lupus (NPSLE) is one of the most common manifestations of human SLE, often causing depression. Interferon-α (IFNα) is a central mediator in disease pathogenesis. Administration of IFNα to patients with chronic viral infections or cancers causes depressive symptoms. Angiotensin-converting enzyme (ACE) is part of the kallikrein–kinin/renin-angiotensin (KKS/RAS) system that regulates many physiological processes, including inflammation, and brain functions. It is known that ACE degrades bradykinin (BK) into inactive peptides. We have previously shown in an *in vitro* model of mouse bone-marrow-derived dendritic cells (BMDC) and human peripheral blood mononuclear cells that captopril (a centrally acting ACE inhibitor-ACEi) suppressed Type I IFN responsive gene (IRG) expression. In this report, we used the MRL/lpr lupus-prone mouse model, an established model to study NPSLE, to determine the *in vivo* effects of captopril on Type I IFN and associated immune responses in the periphery and brain and effects on behavior. Administering captopril to MRL/lpr mice decreased expression of IRGs in brain, spleen and kidney, decreased circulating and tissue IFNα levels, decreased microglial activation (IBA-1 expression) and reduced depressive-like behavior. Serotonin levels that are decreased in depression were increased by captopril treatment. Captopril also reduced autoantibody levels in plasma and immune complex deposition in kidney and brain. Thus, ACEi’s may have potential for therapeutic use for systemic and NPSLE.

## Introduction

Systemic lupus erythematosus (SLE) is a complex systemic autoimmune disease characterized by the loss of tolerance to nuclear antigens, immune complex formation, and inflammation in multiple organs (1). Interferon-α (IFNα) is a central player in lupus pathogenesis (1, 2). Many clinical studies (3, 4) have shown that administration of IFNα, used as a therapeutic for cancer and chronic viral infections, causes the development of depressive symptoms in a high percentage of patients. Major depression is the most common psychiatric presentation of neuropsychiatric lupus (NPSLE) (5, 6). The management of patients with NPSLE continues to be a major therapeutic challenge (7).

Attenuation of the Type I IFN response has been the goal of many recent therapeutic interventions in lupus (8–10). Blocking IFNα or IFN receptor (IFNAR) has been tested in clinical trials with varied outcomes (11, 12). We have shown recently that bradykinins (BK) and captopril [an ACE inhibitor (ACEi)] suppressed Type I IFN responses in murine dendritic cells (DCs) from normal and lupus-prone mice and in human peripheral blood mononuclear cells (PBMC) (13). We also showed that the IFN-suppressive effect of captopril *in vitro* was mediated, in part, via BK receptors (13). Angiotensin-converting enzyme (ACE) is part of the kallikrein–kinin/renin-angiotensin (KKS/RAS) system that regulates many physiological processes, including inflammation, and brain functions (14–17). It is known that ACE degrades bradykinin (BK) to inactive peptides (14, 18) and ACEi’s might help restore the levels. Recent studies have shown that centrally-acting-ACEi’s [that are known to cross the blood-brain barrier (BBB)] decrease microglial activation and restore cognitive deficits in a lupus antibody-mediated (anti-DNA antibodies that cross-react with N-methyl-D-aspartate) neuroinflammation model (19). These antibodies have been found in the brain and cerebrospinal fluid of CNS lupus patients (20, 21). BBB-permeable ACEi’s (captopril and perindopril) have been shown to slow cognitive decline in hypertensive patients with Alzheimer’s disease (22).

In this report, we present the effects of captopril, a centrally-acting-ACEi, on Type I IFN responses, peripheral and CNS inflammatory responses and depressive-like behavior in the MRL/lpr lupus-prone mouse model, in an early intervention short-term study. We show a constitutive IFN signature in the MRL/lpr mice even at an early age (8 weeks) when depressive-like behavior is also present. Systemic or oral administration of captopril (starting at 9 weeks of age) effectively reduced Type I IFN responses, peripheral and CNS inflammation revealed by decreased inflammatory cytokines and reduced microglial activation and reduced depressive-like behavior in the MRL/lpr mice. Plasma serotonin levels, an indicator of depression (23–25), and also a biomarker of kidney disease in lupus (24) was also increased by captopril, suggesting improvement in this behavior and also kidney pathology. Classic clinical markers such as autoantibody levels and immune-complex deposition were also decreased by captopril treatment. We have demonstrated similar effects in the NZB/W F1 IFN-dependent (26) lupus-prone mouse model.

## Materials and Methods

### Mice

MRL/lpr and MRL/wt (MRL/MpJ) female mice were purchased from the Jackson Laboratory (Maine, United States) and used in all experiments starting at 8 weeks (baseline). It has been shown that MRL/lpr mice start to develop autoantibodies beginning at 9 weeks of age; therefore, we started treatment in our cohort at this age. NZB/W F1 female mice were tested at about 20 weeks of age, when autoantibodies have already started to develop (27). All animals were maintained in the animal facility of Temple University, an American Association for the Accreditation of Laboratory Animal Care-accredited facility, following the guidelines of the Institutional Animal Care and Use Committee of Temple University. Three to five mice per treatment group were tested in each set of experiments.

### Captopril Treatment

For systemic administration, MRL/lpr mice were injected with captopril (Sigma Aldrich, Millipore Sigma, MO, United States; 5 mg/kg body weight) intraperitoneally (i.p.) every other day for 2 weeks (19, 28). Control mice were given an equal volume of sterile saline for the same length of time. For oral treatment, MRL/lpr (starting at 9 weeks of age) and NZB/W F1 (starting at 20 weeks of age) mice were given an oral dose of 5 mg/kg of bodyweight captopril (19), 5 days/week until 30 doses were administered by non-forceful feeding. Captopril was dissolved in phosphate buffered saline (PBS) at a concentration such that the maximum volume did not exceed 20 μl per dose. Each mouse was held firmly by the skin around the neck and back of body to gain access to the oral cavity and the compound was administered using a pipette. All the mice typically consumed the entire volume of the captopril or saline. Mice were weighed every week and doses were adjusted. Control mice were given 20 μl of PBS for the same treatment period. Oral treatment by this non-forceful feeding method has been established in our laboratory (29, 30).

### Administration of IFNα via Osmotic Pumps

Recombinant IFNα (5 × 10^5^ U/mouse) was administered to MRL/lpr mice via osmotic pumps (Alzet, CA, United States) that were inserted subcutaneously following sterile procedures. Osmotic pumps were first weighed and primed with recombinant human IFNα (PBL Laboratories, NJ, United States) or PBS for 48 h before surgically inserting into the mice. All the pumps had a reservoir of 200 μL, which pumped 0.25 μl/h over the course of 14 days. In these experiments, captopril was i.p. administered starting 2 days after IFNα pump implantation and mice were euthanized and analyzed after 2 weeks.

### Proteinuria Measurement

Urine was collected weekly and proteinuria was measured by dipstick method (Multistix 10 SG; Siemens, United States). Briefly, mice were placed in individual clean, empty cages to urinate and the urine was collected using a pipette and applied to dipsticks. Color was scored as per the kit instructions (+1 is 30 mg/dl, +2 is 100 mg/dl, +3 is 300 mg/dl, and +4 is 2000 mg/dl).

### Blood Urea Nitrogen Analysis

Mice were anesthetized using isoflurane and blood was collected every 2 weeks by sub-mandibular bleeding and blood urea nitrogen (BUN) was analyzed by dipsticks (Azostix reagent strips, Siemens) per manufacturer’s instructions and scored per chart in the kit and marked as: 1 – 5–15 mg/dl, 2 – 15–25 mg/dl, 3 – 30–40 mg/dl, and 4 – 50–80 mg/dl). Strips were scored by two personnel in a blinded fashion.

### Behavior Analysis

#### Forced Swim Test

Porsolt’s forced-swim test (FST) was used to measure depressive-like behavior using the protocol established by Putterman’s group (31–33). The forced swim test consists of a glass or plastic cylinder, approximately 45 cm high, and 21 cm in diameter, filled 15 cm high with clean water that is maintained at a temperature of 27°C ± 2°C. Animals were placed into the water for 10 min, during which their behavior was video recorded with a GoPro camera and then scored using Boris software. Immobility or floating time and time or latency to become immobile are two parameters used to assess depression-like behaviors (34). Latency to immobility was defined as the time it took until the first period of immobility (floating) that lasted 8 s or longer. The time of immobility in the treated and untreated groups were analyzed during the 9 min of the test, leaving the first minute for acclimation, following the protocol from Putterman’s group (35). The scorers were blinded to the experimental treatment of the mice.

#### Rotarod Test

We also performed a rotarod test (36) or measured locomotor activity to assess locomotor function. The rotarod is used to measure an animal’s motor skills, co-ordination, and balance (37). The test involves a mouse being placed on a horizontally oriented, rotating cylinder (rod) suspended above a cage floor. The rotating cylinder is high enough to induce avoidance of fall but not high enough to injure the animal. Rodents naturally try to stay on the rotating cylinder, or rotarod, and avoid falling to the ground. The length of time that the mouse stays on the rotating rod is a measure of balance, coordination, physical condition, and motor planning. The rotarod test was used in the MRL (wt and lpr) mice to check for locomotor impairment (38).

#### Locomotor Activity Monitoring Test

Lupus-prone mice in the captopril treated cohorts were assessed for locomotor deficits (37) by a locomotor activity monitoring test. Mice were acclimated to the behavioral testing room in their home cages for 30 min prior to initiating the locomotor activity assay, and then placed into a clean, translucent open field chamber (approximately 18” L × 14” W × 8” H) for a 10 min testing session. Activity in the open field was monitored using the AccuScan Home Cage Activity System (Omnitech Electronics, Inc., Columbus, OH, United States), which consists of a cage frame that houses a set of 16 photobeams arranged along the horizontal axis of the testing chamber, and a pair of sensor panels used to detect the beams. Fusion Software (Omnitech Electronics, Inc.) was used to collect data from the sensor panels across multiple Home Cage System Variables in 2 min intervals over the 10 min testing session. At the end of the testing session, animals were removed from the open field chamber and placed into their home cages and returned to the animal housing room.

#### Elevated Plus Maze Test

We used the elevated plus maze test (EPM) to evaluate anxiety-like behavior (39, 40) in the MRL/lpr and the NZB/W F1 mice in the captopril treated cohort. The elevated plus maze consists of a plastic cross standing approximately 52 cm off the ground with two open arms, 45 cm × 10 cm, and two closed arms of the same dimension with 30 cm high walls and 0.5 cm ledges (41). Mice were placed at the center of the four arms and videotaped while they explore the maze for 10 min. The mouse must pass the line of the open platform with all four paws to be considered as an entry into any arm. The duration (in seconds) of time spent in the open arm from the time of entry was recorded. Decreased percent time spent in the open arm indicates a higher level of anxiety-like behavior (42).

### Harvest of Mouse Organs

At the terminal time-point in each experiment, mice were perfused with saline and brain, spleen and kidney were removed and stored at minus 80°C for gene and protein analysis in their respective storage reagents. A portion of fresh spleen tissue was taken and processed for flow cytometric analysis as described elsewhere (43).

### Flow Cytometry

Splenocytes were processed to a single cell suspension and incubated with Fixable Viability Dye (FVD 780, Invitrogen, CA, United States) for 20 min on ice. Cells were washed and mouse Fc blocking antibody (BD Biosciences, CA, United States) was added and incubated on ice for 15 min. Cells were then stained using anti-mouse CD11c antibody (N418 clone; eBioscience, CA, United States) for 30 min on ice, washed and fixed with 2% paraformaldehyde. Intracellular staining for IFNα was performed after permeabilizing the fixed cells in 1x permeabilizing buffer containing saponin (eBioscience) followed by incubation with purified anti-mouse IFNα antibody (Leinco Technologies, MO, United States) for 1 h at room temperature. Cells were washed and secondary antibody tagged to Alexa-488 fluorochrome was added and stained for 20 min. Cells were washed and resuspended in staining buffer and acquired on a BD FACS Canto II cytometer within 24 h. All the data collected were exported and analyzed using FlowJo software version 9 (FlowJo LLC, OR, United States).

### Western Blot

Spleen tissue samples were processed and stored in lysis buffer (Cell Lytic buffer with protease and phosphatase inhibitors added, Thermo-Fisher Scientific, MA, United States) at −80°C. Total protein from the samples were assayed by BCA colorimetric assay. About 30 μg of protein from each sample was loaded in a 4–20% Bis-Tris gel (Bio-Rad, CA, United States) after denaturation at 95°C for 10 min. After proteins from the gel were transferred on to a nitrocellulose membrane, Ponceau staining was performed to make sure that the protein transfer was even. The membrane was then blocked with 2% blocking buffer (non-fat milk in PBS with 0.1% Tween) for 1 h and washed with PBST (PBS + 0.1% Tween). Anti-mouse IFNAR1 was added as primary antibody (Leinco Technologies) in 1:250 dilution in enhancer solution 1 (Millipore). Mouse actin antibody was added as loading control and incubated at 4°C overnight. Membranes were washed with PBST and secondary antibody was added (IR-dye-800-goat anti-mouse secondary antibody diluted 1:10,000). Anti-goat secondary IR dye antibody was used for actin, incubated for 1 h, washed with PBST and scanned in an Odyssey Infrared Imaging System (LI-COR Biosciences, NE, United States) under the 800 nm green channel for protein band visualization. ImageJ software was used to quantitate the band intensities. Band intensities were normalized to actin control.

### IBA-1 Staining

Microscopic examination of the MRL/lpr brains treated with captopril used a standardized immunohistochemistry protocol as described previously (44). Paraffin sections (5 μm) were used for the evaluation of microglial activation using IBA (1:100, Wako Chemicals USA, VA, United States). Primary antibodies were detected by peroxidase using the Envision HRP Labeled Polymer Kit (Vector Laboratories, CA, United States). Samples were imaged under 20x and 40x objective magnification using Olympus BX31 microscope. For each mouse, 4–6 randomly selected fields in the cortex were analyzed.

### Immunofluorescence Staining for C3 and IgG

After perfusion, the brain and kidney tissues were placed in plastic molds in Optimal Cutting Temperature (OCT) compound, placed on dry ice to freeze, and then stored at −80°C. The brain and kidney tissues were sectioned at 10 μm and 6 μm thick slices, respectively, using a Leica CM1860 cryostat (Leica Biosystems, Wetzlar, Germany) and placed on a glass slide for further staining. Brain and kidney tissues were stained for complement 3 (C3) using donkey anti-mouse C3 antibody (Novus Biologicals, CO, United States) or immunoglobulin G (IgG) using Alexa Fluor 488 Goat Anti-Hamster IgG (H&L; Invitrogen, CA, United States). For kidney and brain tissues, 150 μl of conjugated IgG antibody (1:50 dilution in Dako diluent; Agilent, CA, United States) was added to each section and incubated overnight at 4°C. Brain tissues were also stained for microvessels using *Lycopersicon esculentum* (Tomato) lectin antibody conjugated to Texas red (1:500 dilution; Invitrogen). Slides were placed in pre-chilled acetone to fix the tissue sections and washed with TBST (Tris buffered saline with 2% Tween 20). Normal donkey serum diluted to 10% in TBS (Tris buffered saline) was added to each slide and incubated for 1 h at room temperature. About 150 μl of C3 primary antibody was added (1:50 dilution in Dako diluent) to each slide and incubated overnight at 4°C. The following day, slides were washed with TBST and 150 μl of donkey anti-rat Alexa 488 secondary antibody was added (1:500 dilution in normal donkey serum) and incubated for 1 h at room temperature and washed again with TBST. For brain tissue sections, after washing off the secondary antibody, 150 μl of the lectin-tomato (1:500 dilution) was added and incubated overnight at 4°C. Slides were washed with TBST, stained with DAPI, fixed in Prolong Gold anti-fade reagent (Invitrogen) and stored at 4°C protected from light until imaging. All slides were imaged using an Olympus BX31 microscope. Images were exported and the stain intensity was quantified using ImageJ software (NIH). Mean intensity/area was calculated for each region of interest (at least 5 regions for each tissue section from each mouse). Analysis was performed in a blinded fashion to avoid bias in interpretation of the results.

### RNA Extraction and q-PCR

Gene expression in brain, kidney, and spleen samples was analyzed using TaqMan probes and real time quantitative PCR (qPCR). RNA was extracted using TriZol (Invitrogen) following the manufacturers protocol. DNA removal was performed using DNA-free^TM^ Kit (Invitrogen) following the manufacturer’s protocol. Complementary DNA (cDNA) was synthesized with the cDNA archive kit (Life Technologies, CA, United States). qPCR was performed using TaqMan primers and probes for mouse genes Irf7 (Mm00516788_m1), Cxcl10 (Mm00445235), ISG15 (Mm01705338_s1), and ACE (Mm00802048_m1; Applied Biosystems, CA, United States). Cyclophilin was used as reference gene (Mm02342430_g1). ΔΔCt method was used to calculate the fold changes in gene expression by normalizing the values to the control group in each experiment.

### ELISA

Multiplex proinflammatory cytokines and chemokine ELISA kits were bought from Mesoscale Discovery (Rockville, MD, United States) and performed in plasma samples as per the manufacturer’s instructions. IFNα levels in plasma was detected using high sensitivity Verikine IFNα ELISA kit from PBL (PBL Assay Science, NJ, United States). Plasma serotonin levels in the plasma samples were assayed using ELISA kit from Abcam (MA, United States) as per manufacturer’s instructions.

Autoantibodies (anti-dsDNA and anti-chromatin) were measured using methods described elsewhere (43). MRL/lpr and NZB/W F1 mice were bled at baseline (before starting captopril treatment) and 2 weeks after starting treatment and at the end of experiment. Plasma was separated and tested for anti-DNA and anti-chromatin antibodies by ELISA (45). Briefly, polypropylene 96-well plates were coated with chicken erythrocyte-derived chromatin at 3 mg/ml or with calf thymus-derived dsDNA at 2.5 mg/ml in borate-buffered saline (BBS). Plates were coated with poly(L-lysine; 1 mg/ml; Sigma-Aldrich) before coating with Ag for the anti-dsDNA ELISA. Plates were blocked with blocking buffer (3% BSA and 1% Tween 80 in 13 BBS), and plasma samples were diluted 1/250 in BBT (BBS, 0.4% Tween 80, 0.5% BSA) and added in duplicate and incubated overnight at 4°C. Alkaline phosphatase-conjugated goat anti-mouse IgG (Fc γ specific; Jackson ImmunoResearch Laboratories, PA, United States) was used as secondary Ab. Plates were developed using 1 mg/ml para-nitrophenyl phosphate substrate (Sigma-Aldrich) in 0.01 M diethanolamine (pH 9.8). Plasma from old MRL/lpr mouse with high titer of autoantibodies was run as positive control and plasma from C57BL/6 mice was used as negative control.

### Statistical Analysis

Prism software (GraphPad, CA, United States) was used for statistical analysis. Unpaired *t*-test for comparison between two groups was used. Two-way repeated measures ANOVA was used to analyze locomotor assay. *P* < 0.05 were considered significant and marked in the figures as follows: ^∗^*p* < 0.05, ^∗∗^*p* < 0.01, and ^∗∗∗^*p* < 0.001.

## Results

### Young MRL/lpr Mice Present a Constitutive IFN Signature

**FIGURE 1:**
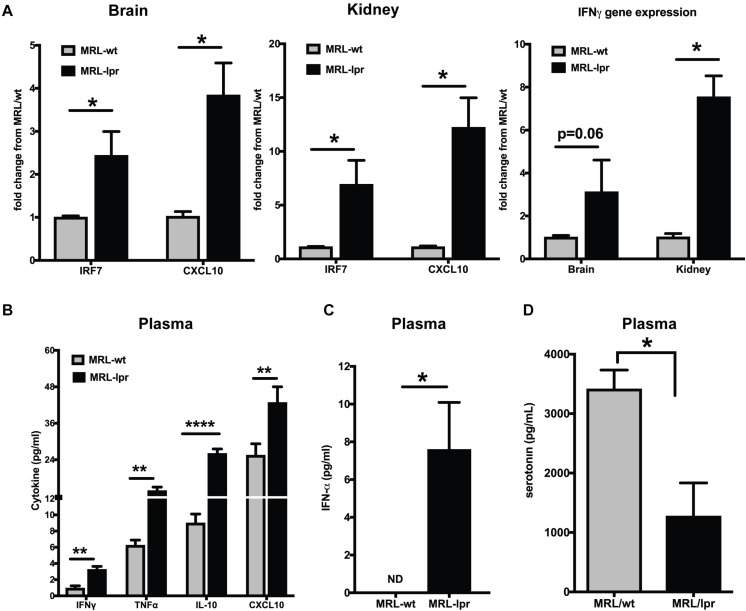
Young MRL/lpr mice present a constitutive IFN signature. **(A)** Gene expression of IRF7, CXCL10, and IFNγ in brain and kidney was analyzed by Taqman qPCR. ΔΔCt method was used to calculate the fold changes using MRl/wt mice as controls. Cyclophilin gene was used for normalizing gene expression. **(B)** Multiplex cytokine analysis was done using the MSD ELISA kit. Key cytokines >5 pg/ml that are significantly different between the MRL/lpr and MRL/wt mice are represented. **(C)** Plasma IFNα levels were measured using high sensitivity Verikine ELISA kit from PBL. **(D)** Serotonin levels were measured in the plasma; levels in MRL/lpr were significantly low. Unpaired *t* test statistical analysis was performed and *p* < 0.05 was considered significant; *p < 0.05, **p < 0.01, and ****p < 0.0001; *n* = 3–5 mice/group.

The MRL/lpr mouse strain is one of the best-studied spontaneous models for lupus (46). This mouse strain is homozygous for the lymphoproliferation mutation (Fas^*lpr*^) and exhibits autoimmune disease that closely resembles human SLE. The MRL/MpJTnfrsf6^*lpr*^ (MRL/lpr) mice and MRL/MpJ+/+(MRL/wt) congenic control mice differ in the onset of lupus-like manifestations by about 3–4 months (35). We found constitutively increased interferon responsive genes (IRGs) in the brain and kidney of young MRL/lpr mice as compared to the MRL/wt (8-week-old), measured by qPCR (Figure 1A). IRF7, the master regulator of Type I IFN (47), and CXCL10, an important biomarker of lupus disease (48), were increased in the brain and kidney of MRL/lpr mice compared to MRL/wt controls. IFN γ levels have been shown to be increased in MRL/lpr mice at late stages of the disease (49). We found increased IFNγ gene expression in the brain and kidney of 8-week-old MRL/lpr mice compared to age matched wild type mice (MRL/wt; Figure 1A). Circulating IFNγ levels, albeit low, was also significantly increased in the MRL/lpr (Figure 1B). CXCL10 (IP10) levels were also high in the plasma, as were levels of TNFα and IL-10 (Figure 1B). Circulating IFNα levels were also significantly high in MRL/lpr mice compared to MRL/wt mice (Figure 1C). Decreased levels of the neurotransmitter serotonin have been implicated in depression (24, 50). We found decreased serotonin levels in plasma of young MRL/lpr mice compared to MRL/wt mice (Figure 1D) and the same mice showed increased depressive-like behavior by Porsolt’s forced swim test (51) (Supplementary Figure S1).

### Captopril Reduces IFN-Stimulated Genes in the Brain and the Periphery in Lupus-Prone Mice

**FIGURE 2:**
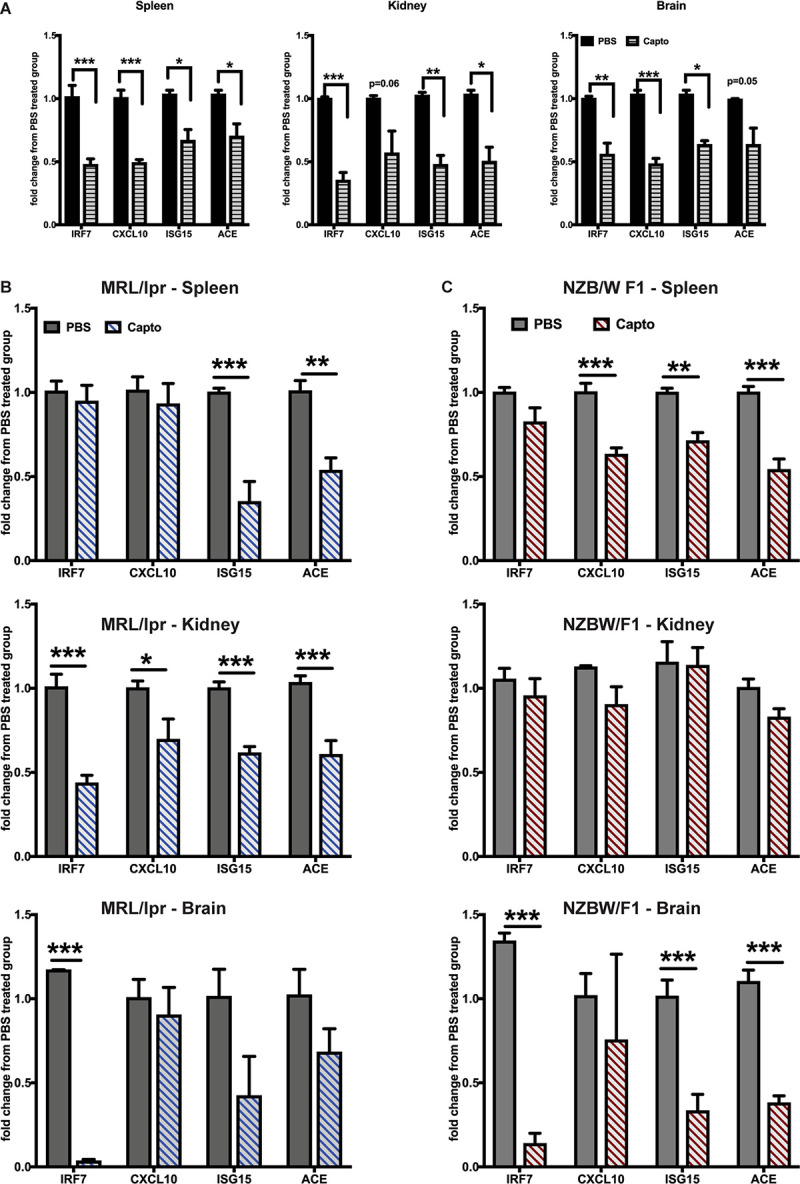
Captopril reduces IRGs in brain, spleen and kidney in lupus-prone mice. **(A)** MRL/lpr mice were treated with captopril (i.p. 5 mg/kg body wt.) for 2 weeks and analyzed for IRGs in spleen, kidney and brain by Taqman qPCR. IRG expression in the spleen, kidney and brain was analyzed after oral captopril treatment in MRL/lpr **(B)** and NZB/W F1 mice **(C)**. ΔΔCt method was used to calculate the fold changes in gene expression using PBS-treated mice as a control group. Cyclophilin gene was used for normalizing gene expression. *N* = 3–5 mice/group; Unpaired *t* test statistical analysis was performed and *p* < 0.05 was considered significant; *p < 0.05, **p < 0.01, and ***p < 0.001.

The effects of ACEi’s have been recently demonstrated using an anti-NMDAR antibody (a subset of lupus anti-DNA antibody) induced neuroinflammation model (19). Administering captopril via i.p. (Figure 2A) or oral route (Figures 2B,C) in the spontaneous lupus-prone mice, decreased IRGs in the brain, spleen and kidney in both MRL/lpr, and the interferon-dependent NZB/W F1 lupus-prone mouse models. ACE gene expression was reduced in all tissues with captopril treatment (Figures 2B,C), suggesting that the treatment affected ACE expression directly at the transcriptional level in both strains of mice. In the MRL/lpr mice, systemic administration of captopril (Figure 2A), however, had a stronger effect in all tissues, while oral administration had significant effects only in the kidney for all the genes tested, suggesting that drug absorption and availability in the different tissues may vary and so the responses. There were differences in the extent of suppression of IRGs in the two strains of mice (Figures 2B,C). The differences in the response in the two strains could be, in part, due to the intrinsic differences in the IFN levels/response, the NZB/W F1 being a validated IFN-dependent model (26). The treatment in the MRL/lpr was initiated at 9 weeks of age (just at the start of autoantibody production in this strain), while the NZB/W F1 were studied at 20 weeks of age (autoantibodies begin to form from about 16 weeks in this strain). Captopril efficiently decreased IRGs in both situations, suggesting that the drug can be effective early in the course of the disease.

### Captopril Administration Reduces IFNα Levels and Inflammatory Cytokines in Plasma and Spleen of MRL/lpr Mice

**FIGURE 3:**
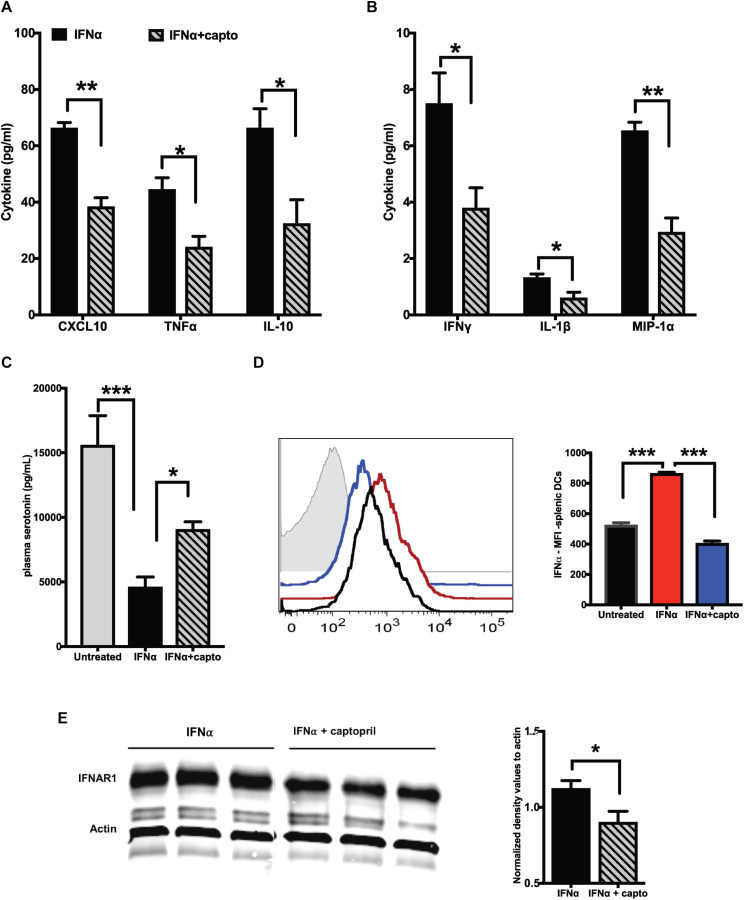
Captopril reduces IFN responsive protein expression. Plasma from MRL/lpr mice induced with exogenous IFNα and treated with captopril for 2 weeks were analyzed for cytokines and chemokines using MSD multiplex ELISA kit. Significantly different cytokines and chemokines are represented, **(A)** >10 pg/ml and **(B)** <10 pg/ml (*n* = 4/group). **(C)** Serotonin levels in plasma of IFNα induced MRL/lpr mice was measured using ELISA. **(D)** Splenocytes of IFNα treated MRL/lpr mice were harvested, stained for intracellular IFNα, gated and analyzed on CD11c + cells. Median fluorescence intensities (MFI) are represented as histograms on the left and the corresponding colors for the different groups indicated in the bar graph on the right (*n* = 4/group). Unstained control is shown in gray in the histogram. **(E)** Spleen lysates of exogenous IFNα treated MRL/lpr mice were analyzed by western blotting to detect IFNAR1 protein. Actin was used a loading control. Band intensities were analyzed using ImageJ software and normalized values are represented in the graph. Unpaired *t* test statistical analysis was performed and *p* < 0.05 was considered significant; *p < 0.05, **p < 0.01, and ***p < 0.001.

We next analyzed if an exogenous IFNα induced response can be suppressed by captopril in the MRL/lpr mice. We administered recombinant IFNα as described in the Methods section, followed by systemic captopril administration for 2 weeks. Captopril significantly reduced the proinflammatory cytokines and chemokines as shown in Figures 3A,B. IL-10 was also reduced by captopril (Figure 3A). To further confirm the effects of captopril on IFNα production, we analyzed splenic DCs by flow cytometry (Figure 3D). IFNα treatment increased IFNα production in the splenic DCs, as shown by the increase in mean fluorescence intensity (MFI) of IFNα staining. Captopril significantly lowered the intensity, suggesting that captopril can help reduce, not only the response to IFNα, but also the production of IFNα *per se*. IFNα binds to its receptor IFNAR1 and IFNAR2 to bring about further signaling and response (52). We also analyzed if captopril can reduce IFNAR1 protein expression in our cohort of MRL/lpr mice that was treated with exogenous IFNα. As shown in Figure 3E, captopril decreased IFNAR1 protein expression in the spleen. We showed constitutively decreased plasma serotonin levels in the young MRL/lpr mice (Figure 1D). We measured serotonin levels after IFNα treatment and found that IFNα significantly decreased plasma serotonin levels and captopril treatment could help reverse this effect (Figure 3C).

### Captopril Administration Reduces Microglial Activation in the Brain of MRL/lpr Mice

**FIGURE 4:**
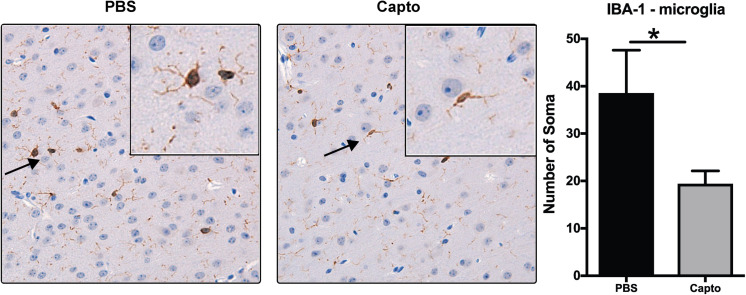
Captopril reduces microglial activation in the brain. Young MRL/lpr mice treated with captopril (i.p.) were perfused at end of treatment and brain tissue was harvested, paraffin embedded and analyzed for IBA-1 expression by chromagen (DAB) staining and IBA-1 staining in the PBS and captopril treated groups. IBA-1 staining of brain microglia shows more activation in PBS-treated MRL/lpr mice than in captopril-treated mice. Inset shows 40x magnification of the cortex region showing activated microglia with retracted processes and bigger cell bodies in PBS- treated mice vs. less activated microglia in captopril-treated mice. Cells stained for IBA-1 (brown) were counted in each of 6 fields on each slide for each mouse and averaged; *n* = 3–5 mice/group. Slides were coded and read in a blinded fashion to avoid any bias in interpretation. Unpaired *t* test statistical analysis was performed and *p* < 0.05 was considered significant; *p < 0.05.

To study the effects of captopril on microglia in the MRL/lpr spontaneous mouse model, we analyzed the expression of IBA-1 as a marker of microglial activation (Figure 4). Mice receiving PBS only had intense staining for IBA-1 in bigger bodies (soma) with lesser/shorter ramifications as compared to captopril treated group that had less intense staining.

### Captopril Reduces Depressive-Like and Anxiety-Like Behavior in Lupus-Prone Mice

**FIGURE 5:**
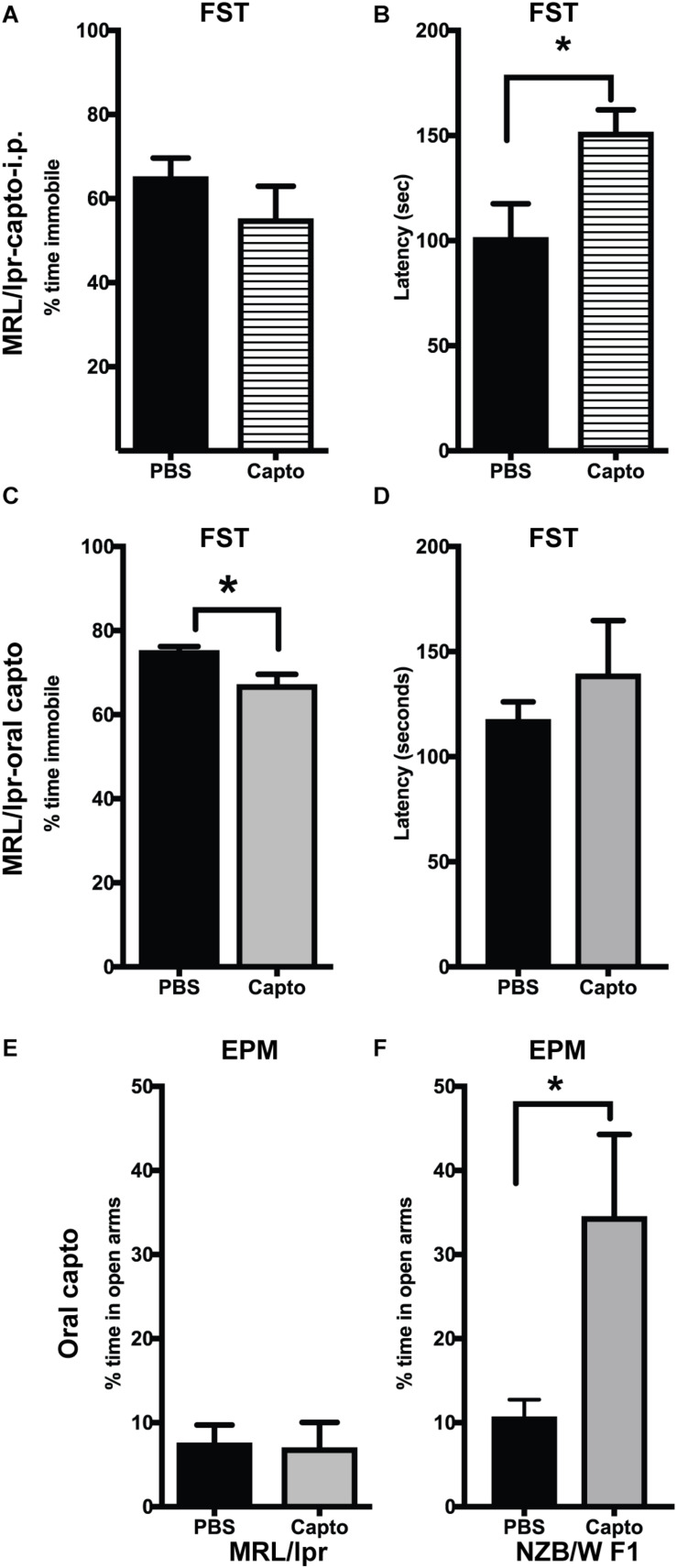
Captopril suppresses depression-like and anxiety-like behavior in lupus-prone mice. The forced swim test was performed in MRL/lpr mice after systemic captopril administration **(A,B)** and oral captopril administration **(C,D)** as described in the Methods section. Percent time immobile is represented in **(A,C)**. Average latency to immobility (in seconds) is represented in **(B,D)**. Captopril mice showed shorter time of immobility and longer latency indicating less depressive behavior than the untreated controls. Anxiety-like behavior was measured by EPM in the MRL/lpr **(E)** and NZB/W F1 **(F)** mice. Percent time spent in the open arms is shown. Captopril treated NZB/W F1 mice spent more time in open arms indicating less anxious behavior compared to PBS treated controls. Data are shown as mean + SE. Students *t* test *p < 0.05, *n* = 6/group.

MRL/lpr mice have been demonstrated to have depressive-like behavior at an early age (8 weeks) (33), with no defect in locomotor function. We confirmed these effects in our cohort using the forced swim test. Immobility was significantly higher in the MRL/lpr mice compared with MRL/wt mouse controls (Supplementary Figure S1). Systemic administration of captopril for 2 weeks reduced depressive-like behavior in the MRL/lpr spontaneous lupus-prone mice (Figures 5A,B). We observed a significant increase in latency to become immobile in the captopril-treated group compared with the PBS-treated mice (Figure 5B). When we extended the treatment time in the oral captopril group to 1 month (Figures 5C,D), the effect on the time of immobility became significant (Figure 5C). There was no difference in the locomotor function in this group (Supplementary Figure S2A), indicating that the differences in the FST is not due to a defect in locomotion, but rather an indication of despair (53). We also found that captopril-treated mice trended to increase the time to latency in the IFNα treated group (Supplementary Figure S2B). A parallel comparison in the NZB/W F1 mice showed that captopril increased the latency to immobility in this strain (Supplementary Figure S2C); however, the values did not reach statistical significance. Anxiety-like behavior has been shown to be related to increased IFNα in the NZB/W F1 mice (54). MRL/lpr mice develop anxiety-like behavior after 10 weeks (31, 35). We analyzed anxiety-like behavior by EPM test at the end of the experimental time-point (13 weeks in the MRL/lpr mice and about 25 weeks in the NZB/W F1 mice) in the oral captopril-treated MRL/lpr and NZB/W F1 mice. Both captopril-treated and untreated MRL/lpr mice showed lesser amounts of time in the open arms in the EPM test suggesting an anxiety-like behavior and there was no effect of captopril on this behavior (Figure 5E). Untreated (PBS) NZB/W F1 mice also showed lesser time in the open arms, while captopril reversed this effect in these lupus-prone mice; the captopril-treated mice spent significantly more time in the open arms compared to the PBS-treated group (Figure 5F).

### Oral Captopril Administration Reduces BUN and Autoantibody Levels in MRL/lpr Mice

**FIGURE 6:**
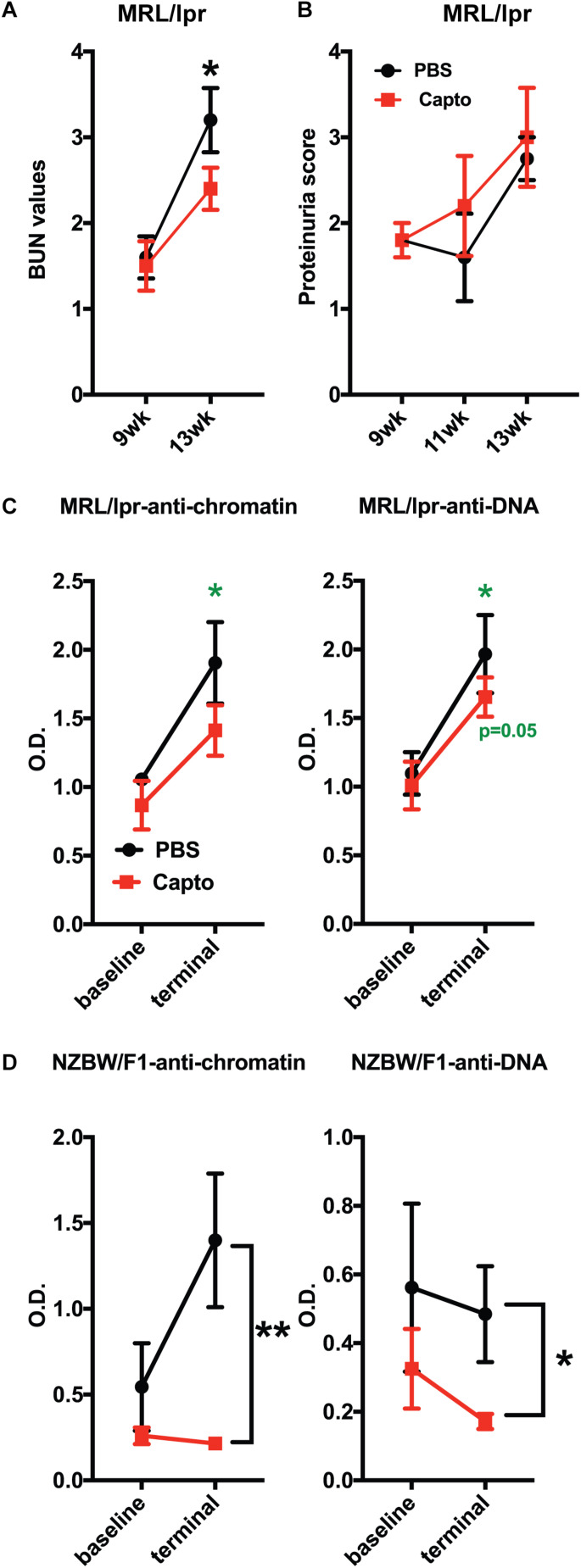
Oral captopril reduces BUN and autoantibody levels in lupus-prone mice. **(A)** MRL/lpr mice orally treated with captopril for about a month and blood was assessed for BUN using Bayer’s dipstick method as per manufacturer’s protocol and scored as 1 (5–15 mg/dL), 2 (15–26 mg/dL), 3 (30–40 mg/dL), and 4 (50–80 mg/dL), as indicated by the color intensity. **(B)** Proteinuria was analyzed using the Multistix dipstick method and color intensities were scored as per manufacturer’s instructions 1-; 2-; 3-; 4. Captopril-treated MRL/lpr (*n* = 5/group); **(C)** and NZB/W F1 (*n* = 8/group); **(D)** mice were analyzed for anti-dsDNA and anti-chromatin autoantibodies. O.D.’s were plotted and compared between the treated and untreated groups. The *p* values shown in green are comparisons before and after treatment in that group using paired Student’s *t* test. *p < 0.05 and **p < 0.01.

Patients with lupus nephritis show high BUN values of >12 mg/dl (55). We tested (BUN) levels in MRL/lpr mice using BUN strips. BUN was performed at baseline (before treatment was started) and every 2 weeks until the end of the experiment (Figure 6A). The baseline values (9 weeks) for both groups were close in range. There was a significant decrease in the BUN (*p* < 0.05) after 1 month of oral captopril treatment, as compared to the control group. These results suggest that captopril can decrease BUN in MRL/lpr mice and may help reduce kidney pathology. However, there was no change in the proteinuria between the treated and untreated groups (Figure 6B). Autoantibodies against anti-dsDNA and anti-chromatin were analyzed in both MRL/lpr and NZB/W F1 mice that were treated with oral captopril. Captopril treatment did not alter the autoantibody levels in the MRL/lpr mice (Figures 6C,D). Both anti-DNA and anti-chromatin antibody levels were decreased in the NZB/W F1 mice with captopril treatment (Figures 6E,F) suggesting that different lupus mouse models respond differently to captopril.

### Captopril Administration Reduces C3 and IgG Expression in MRL/lpr Kidney and Brain

**FIGURE 7:**
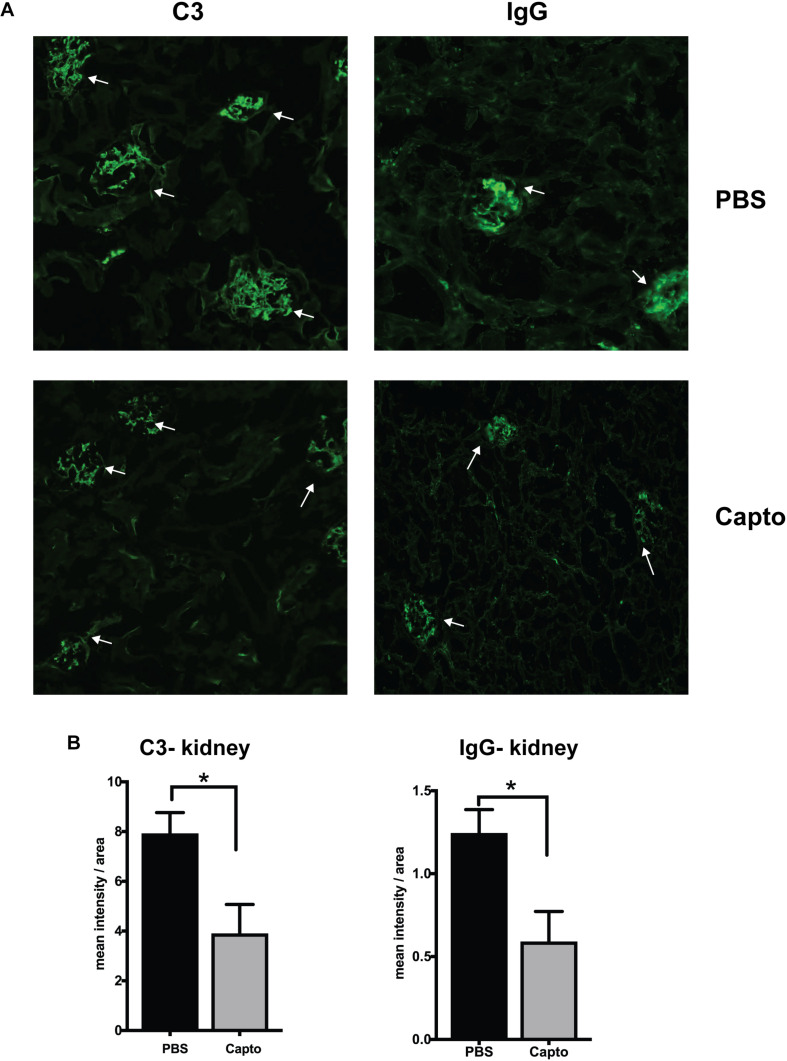
Oral captopril reduces IgG and complement deposition in the kidney. Frozen kidney sections from MRL/lpr mice treated with oral captopril were analyzed for C3 complement and IgG deposition by immunofluorescence staining. **(A)** 200X magnified image shows glomeruli (indicated by arrows) stained more intense with C3 or IgG in the untreated (PBS) group as compared to less intense staining in the captopril-treated group. **(B)** Numerical values of stain intensities are represented in the bar graphs (*n* = 5/group). Unpaired *t* test statistical analysis was performed and *p* < 0.05 was considered significant; *p < 0.05.

**FIGURE 8:**
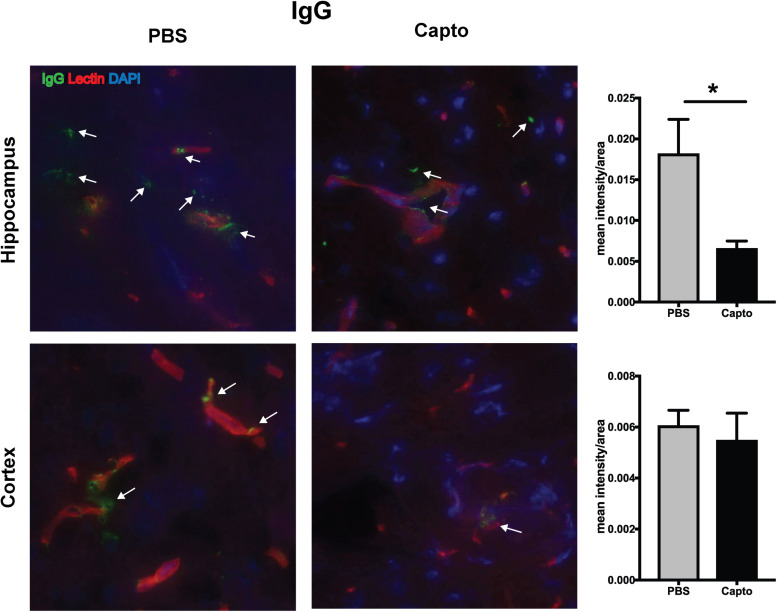
Oral captopril reduces IgG deposition in the brain. Frozen brains from captopril- treated and control MRL/lpr mice were sagitally sectioned and stained for IgG (FITC), tomato lectin (Texas red) for microvessels and DAPI and analyzed in the cortex and hippocampal regions. Representative images (400x magnification) show increased staining for IgG (indicated by arrows) in the hippocampal region of untreated mice than captopril-treated mice (*n* = 5/group). Staining intensity was quantified by ImageJ and unpaired t statistical analysis was performed, *p < 0.05.

The deposition of complement components and immune complexes in the glomeruli is considered to be a hallmark of lupus (56). Immune-complex deposition in the MRL/lpr kidney was analyzed by immunofluorescence staining for C3. As expected, there was prominent deposition of C3 and IgG (Figure 7A) in the glomeruli. Captopril treatment significantly decreased in the levels of C3 deposition in the kidney (Figures 7A,B). IgG and C3 deposition were also analyzed in the cortex and the hippocampal regions of the brain (Figure 8 and Supplementary Figure S3). Captopril reduced IgG and C3 deposition in the hippocampal region (Figure 8 and Supplementary Figure S3, respectively), that was statistically significant for IgG expression than C3. There was diminished signal in the captopril-treated mice brains for IgG and C3 in the cortex region also (Figure 8 and Supplementary Figure S3).

## Discussion

In this short-term study, using both MRL/lpr and NZB/W F1 lupus-prone mouse models, we show that captopril, a BBB-crossing, centrally acting ACEi, effectively reduces IFN responses, peripheral and neuroinflammation and autoantibody levels, when administered at an early disease stage. The decrease in depressive-like behavior responses and decrease in the immune-complex deposition in the brain and kidney with a short-term oral captopril treatment in the MRL/lpr mice (a well-studied NPSLE model) indicates that ACEi’s can be potential drugs that could be repurposed to treat systemic lupus as well as NPSLE.

Interferon-α signatures have been demonstrated in both lupus-prone models in the periphery (1, 9, 10, 57). IFN signatures in the infiltrating cells of the choroid plexus region of the brain in the MRL/lpr mice have only been recently analyzed by Putterman’s group; they have also shown depressive-like behavior in this model (58). We have shown a constitutive increase in the IRGs in total brain lysates and in the kidney in the young MRL/lpr mice at 8 weeks of age, especially the key genes IRF7 and CXCL10. Whether the IFN signature is intrinsic to the brain is still under investigation (58, 59). IRF7 is a key regulator of Type I IFN responses (47, 60). CXCL10 has been shown to be increased in mouse models and lupus patients and is an important biomarker of lupus (48, 61–64). The IFN signature (excessive production/response to Type I IFN) has been shown to be higher in pediatric lupus (65) and during active SLE (57), highlighting IFN as an early therapeutic target. Recent studies indicate that neuropsychiatric symptoms, especially depression and anxiety, may be common comorbidities of childhood-onset of SLE (66, 67). Increased IFNα levels have been shown in the CSF of NPSLE patients (68). IFNα is known to reduce serotonin by skewing the tryptophan-kyneurinine pathway (69). Circulating serotonin levels have been shown to be indicative of kidney disease in lupus (24) as well as an indicator of depression (23, 70). We found decreased plasma serotonin in MRL/lpr mice compared to MRL/wt suggesting a correlation with depressive-like behavior. Our data support the idea that IFNα could be the primary cause of inflammation in the brain and associated depressive-like behavior (71). Our findings clearly suggest that IFNα has an important role in neuropsychiatric behavior in lupus. Genetic studies have shown that excess IFNα in the brain may be one of the causes for anxiety-like behavior in the NZB/W F1 lupus-prone mouse model (54). We did find that captopril treatment improved anxiety-like behavior in NZB/W F1 mice. The effect of captopril on depressive-like behavior in the NZB/W F1 mice was, however, not as significant compared to the effects on the MRL/lpr mice. We did not observe a big difference in cognitive functions (memory; assessed by Y-maze test, data not shown) or anxiety in the spontaneous or the IFNα induced models of MRL/lpr mice with captopril treatment. Putterman’s group has also shown depression is the major behavior characteristic in the MRL/lpr mouse model at the age that we studied (33). This suggests that NPSLE-like behavior characteristics may be different in different lupus mouse models and the ability of captopril to affect behavior patterns may also differ.

Therapeutic approaches for lupus to attenuate IFN responses have focused on agents that block Type I IFN/IFN receptor directly (10, 72) or IFN generated via TLR ligands as TLR7/9 (73, 74). The complexity of Type I IFN production/regulation is continuing to evolve (75). NZB/W F1 mice is validated as an IFN-dependent model (26), while there are controversial reports on IFN effects in the MRL/lpr mice (76, 77). Nonetheless, most evidence points to IFNα contributing to lupus pathogenesis (78). A very recent study to block IFNAR did not reduce NPSLE-like characteristics in the MRL/lpr mouse model (78). This may not indicate that IFNα is not playing a role, but rather the ability of the IFNAR antibodies to reach the brain and affect the CNS. Our recent work (13) shows that candidates of the KKS (BK, klk) and ACEi’s can suppress Type I IFN responses in mice and humans *in vitro*. Several studies have investigated the effects of ACEi’s in systemic lupus (79) either alone or as combination therapy (28). The effect of ACEi’s, especially those that can cross the BBB, on microglial activation and cognitive effects have been demonstrated recently (19). Our short-term study on the effects of the centrally acting, BBB-crossing ACEi, captopril, shows definitive effects of the drug in the brain and the periphery, especially on suppressing IFN responses.

Both centrally acting (captopril, perindopril, lisinopril, and ramipril) and non-centrally acting (enalapril, imidapril) ACEi’s have been tested in lupus mice to analyze effects on kidney disease and peripheral immune responses (80–83). About 30 mg/kg body weight of captopril dissolved in drinking water has been tested in the MRL/lpr and NZB/W F1 mice (84). To our knowledge this is the first report to study the effects of captopril (5 mg/kg body weight) via a non-forceful feeding method and precise oral administration of the drug, and also assess the effects of ACEi on CNS immune responses (neuroinflammation and behavior) in spontaneous lupus-prone mouse models. Our analysis in the spontaneous MRL/lpr and the NZB/W F1 models shows that oral captopril decreased IFN and other inflammatory responses in the periphery and the CNS.

The effects of ACEi’s have been very well described to act mostly via increasing the bioavailability of BK (18). We have earlier shown in murine bone-marrow-derived dendritic cells (BMDCs) that suppression of the IFN response by BK may be brought about, in part, via prostaglandins (13). We hypothesize that ACEi’s potentiate bradykinin-mediated suppression of IFN responses *in vivo* through prostaglandins. In this study, we found that ACEi actually decreased IFNAR expression in MRL/lpr splenocytes *in vivo*, which is well upstream of IFN signaling. Type I IFNs follow an autocrine positive feedback loop via the IFNAR (52). Decreased IFNα production may also affect decreased expression of the receptors. The mechanistic aspects of ACEi and IFN pathways need to be studied in more detail.

Recent studies have shown that microglia are important players in neuroinflammation (19, 85). In a collaborative study using a lupus-antibody-mediated neuroinflammation model, we have shown that ACEi’s prevented microglial activation and restored memory function (19). We have demonstrated in the MRL/lpr mice that captopril not only decreased microglial activation, but also immune-complex deposition in the brain (complement C3 and IgG).

This report demonstrates that captopril (a BBB-crossing ACEi) reduced IRG expression in the brain, spleen and kidney, decreased neuroinflammation and reduced depressive-like behavior in the MRL/lpr lupus-prone mouse model. Our finding of an increased IFN signature in the brain and kidney of MRL mice at a very early stage of the disease is important for understanding of NPSLE pathogenesis. Observations of suppression of IFN response and behavior in the NZB/W F1 mouse model shows that captopril’s effects may be different in the different lupus-prone mouse strains. Our data showing the ability of captopril (a BBB-permeable ACEi) to inhibit the IFN signature also provides a pathway for novel therapeutic intervention for NPSLE. While captopril reduced microglial activation, enalapril (a BBB-non-permeable ACEi) did not, in the lupus antibody induced neuroinflammation model (19). The therapeutic efficacy of captopril has been demonstrated by the alteration of platelet function (86). A study in a small group of cardiovascular patients showed that captopril was effective in attenuating platelet aggregation while enalapril did not (87). Studies in mice and humans have shown that enalapril, although BBB non-permeable, is neuroprotective (88–90). ACEi’s are widely prescribed, well-tolerated, FDA-approved drugs for several cardiovascular indications (91, 92) with rare side effects. Understanding the effects and mechanism of action of other ACEi’s in the brain (93) is an important future direction, as this will help expand the different ACEi’s that could be used for treatment of CNS lupus (94). Our observations in this report are novel and suggest another pathway of action of ACEi’s via the IFN system that warrants further investigation, not only for lupus treatment, but also for other IFN-regulated diseases.

## Data Availability Statement

The raw data supporting the conclusions of this article will be made available by the authors, without undue reservation.

## Ethics Statement

The animal study was reviewed and approved by Institutional Animal Care and Use Committee of Temple University.

## Author Contributions

CN: mouse colony, oral drug administration, tissue harvest, BUN and proteinuria analysis, autoantibody and serotonin elisas, and behavior assays. CL: RNA extraction, PCR, and flow cytometry. MH: behavior studies and analysis of data and histology. SK: immunofluorescence staining and imaging. TP: tissue processing, western blot and histology image analysis, and mice injections. AS: mice injections, oral administration, and tissue harvest. MW: histology. VZ-R: mice injections and harvest. NF: IBA-1 image analysis. XS: help with behavior studies. EU: expert advice for behavior studies and manuscript writing. YP: expert advice on histology and manuscript writing. US: conception and design of experiments, analysis of data, and manuscript writing. All authors contributed to the article and approved the submitted version.

## Conflict of Interest

The authors declare that the research was conducted in the absence of any commercial or financial relationships that could be construed as a potential conflict of interest.

## Supplementary Material

FIGURE S1 Young MRL/wt and MRL/lpr mice (8-week-old) were compared in **(A)** Forced swim test to assess depressive-like behavior. Percent time immobile for each group is represented. MRL/lpr mice were immobile for a significantly greater percent of time than MRL/wt. **(B)** Rotarod test to assess locomotor function. Average latency time (seconds) is represented. No difference in the latency time in both groups suggests that the locomotor function is normal in both strains of mice. Data are mean + SE. Students *t* test ^∗^*p* < 0.05. *n* = 3/grp.

FIGURE S2 **(A)** Locomotor function was tested using a locomotor activity monitoring test as described in the Methods section in MRL/lpr mice treated with PBS or captopril. Mean ambulatory activity counts across a 10 min test (in 2 min bins) is represented. Two-way repeated measures ANOVA was used as the statistic to compare the two groups. No significant difference was observed between the two groups of mice suggesting that locomotor function is normal. **(B)** FST was performed in MRL/lpr mice treated with IFNα and then with captopril. Time to latency (bar graph on the right) was increased in the captopril-treated group as compared to IFNα alone. **(C)** NZB/W F1 mice treated with captopril were analyzed for depressive-like behavior by FST. No difference in time of immobility or latency to immobility was observed between the treated and untreated groups.

FIGURE S3 C3 deposition in MRL/lpr brain. Frozen brains from captopril-treated and control MRL/lpr mice were sagitally sectioned, stained for C3 (Alexa 488) and tomato lectin (Texas red) for microvessels, and analyzed in the cortex and hippocampal regions. Representative images (400x magnification) are shown for C3 in the cortex and hippocampal regions (indicated by arrows) of untreated mice and captopril-treated mice. Staining intensity was quantified by ImageJ. No significant difference in C3 staining was found between the treated and untreated mice.
