# Supplementary figures and images for: Centrally Acting Angiotensin-Converting Enzyme Inhibitor Suppresses Type I Interferon Responses and Decreases Inflammation in the Periphery and the CNS in Lupus-Prone Mice
Source: Front Immunol. 2020 Sep 15;11:573677. doi: 10.3389/fimmu.2020.573677 (PMC7522287; doi:10.3389/fimmu.2020.573677)

**A****FST**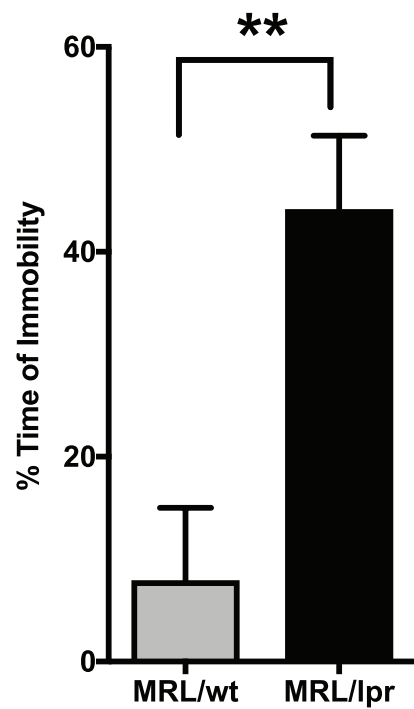**B****Rotarod**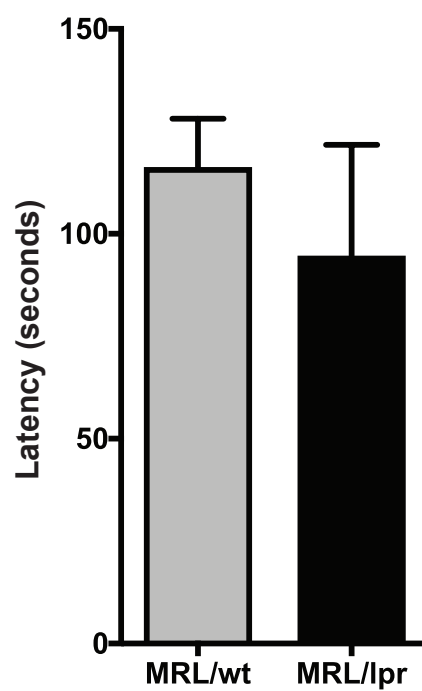

Supplemental Figure 1

Supplement: FIGURE S1 — Young MRL/wt and MRL/lpr mice (8-week-old) were compared in (A) Forced swim test to assess depressive-like behavior. Percent time immobile for each group is represented. MRL/lpr mice were immobile for a significantly greater percent of time than MRL/wt. (B) Rotarod test to assess locomotor function. Average latency time (seconds) is represented. No difference in the latency time in both groups suggests that the locomotor function is normal in both strains of mice. Data are mean + SE. Students t test ∗p < 0.05. n = 3/grp. [file Data_Sheet_1.PDF]

Supplemental Figure 2

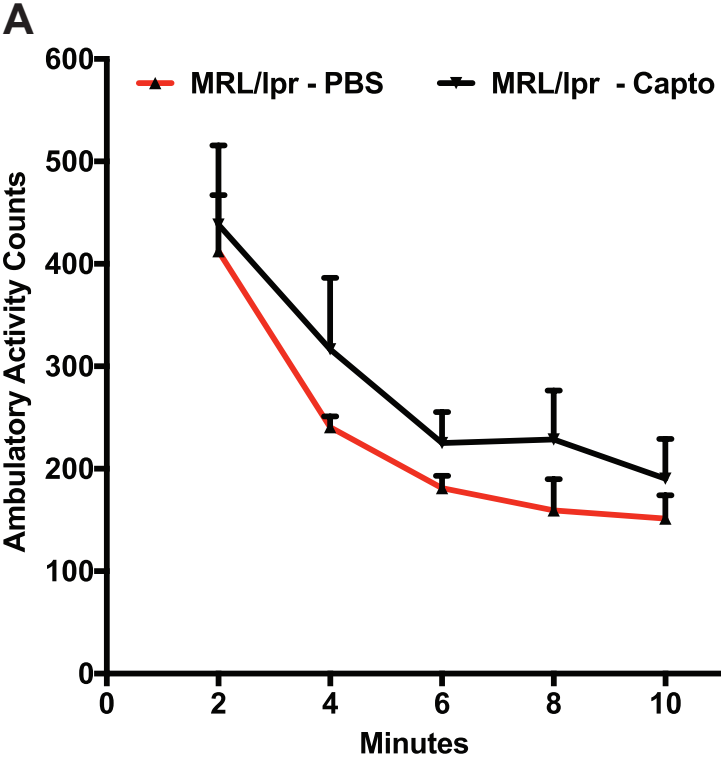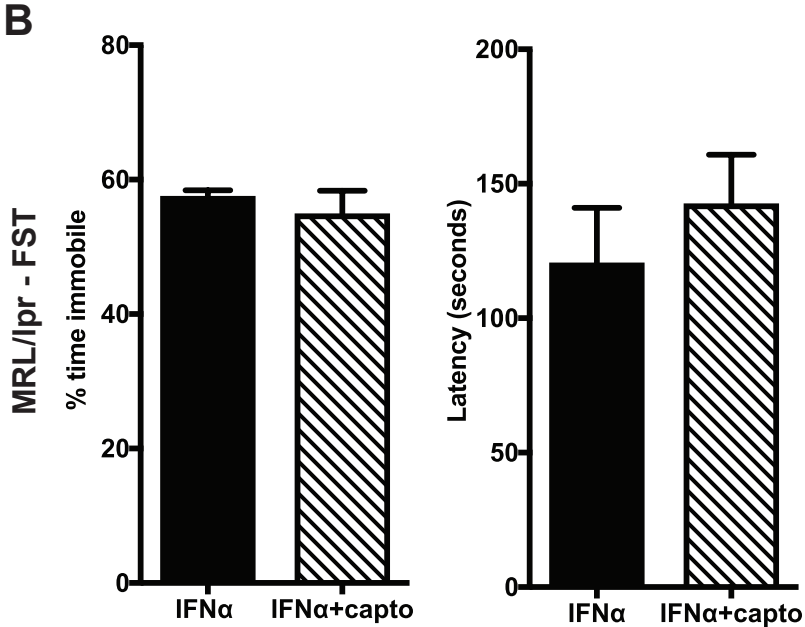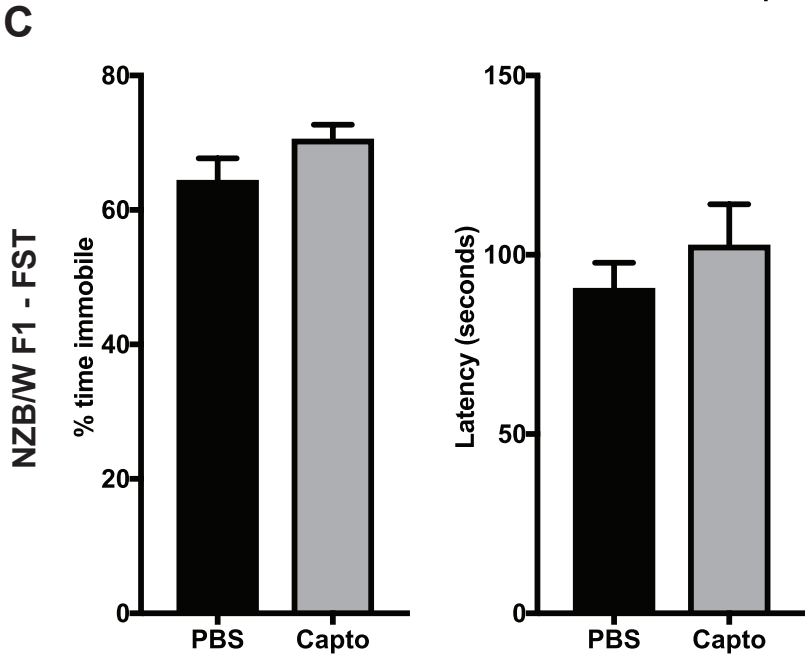

Supplement: FIGURE S2 — (A) Locomotor function was tested using a locomotor activity monitoring test as described in the Methods section in MRL/lpr mice treated with PBS or captopril. Mean ambulatory activity counts across a 10 min test (in 2 min bins) is represented. Two-way repeated measures ANOVA was used as the statistic to compare the two groups. No significant difference was observed between the two groups of mice suggesting that locomotor function is normal. (B) FST was performed in MRL/lpr mice treated with IFNα and then with captopril. Time to latency (bar graph on the right) was increased in the captopril-treated group as compared to IFNα alone. (C) NZB/W F1 mice treated with captopril were analyzed for depressive-like behavior by FST. No difference in time of immobility or latency to immobility was observed between the treated and untreated groups. [file Data_Sheet_2.PDF]

C3

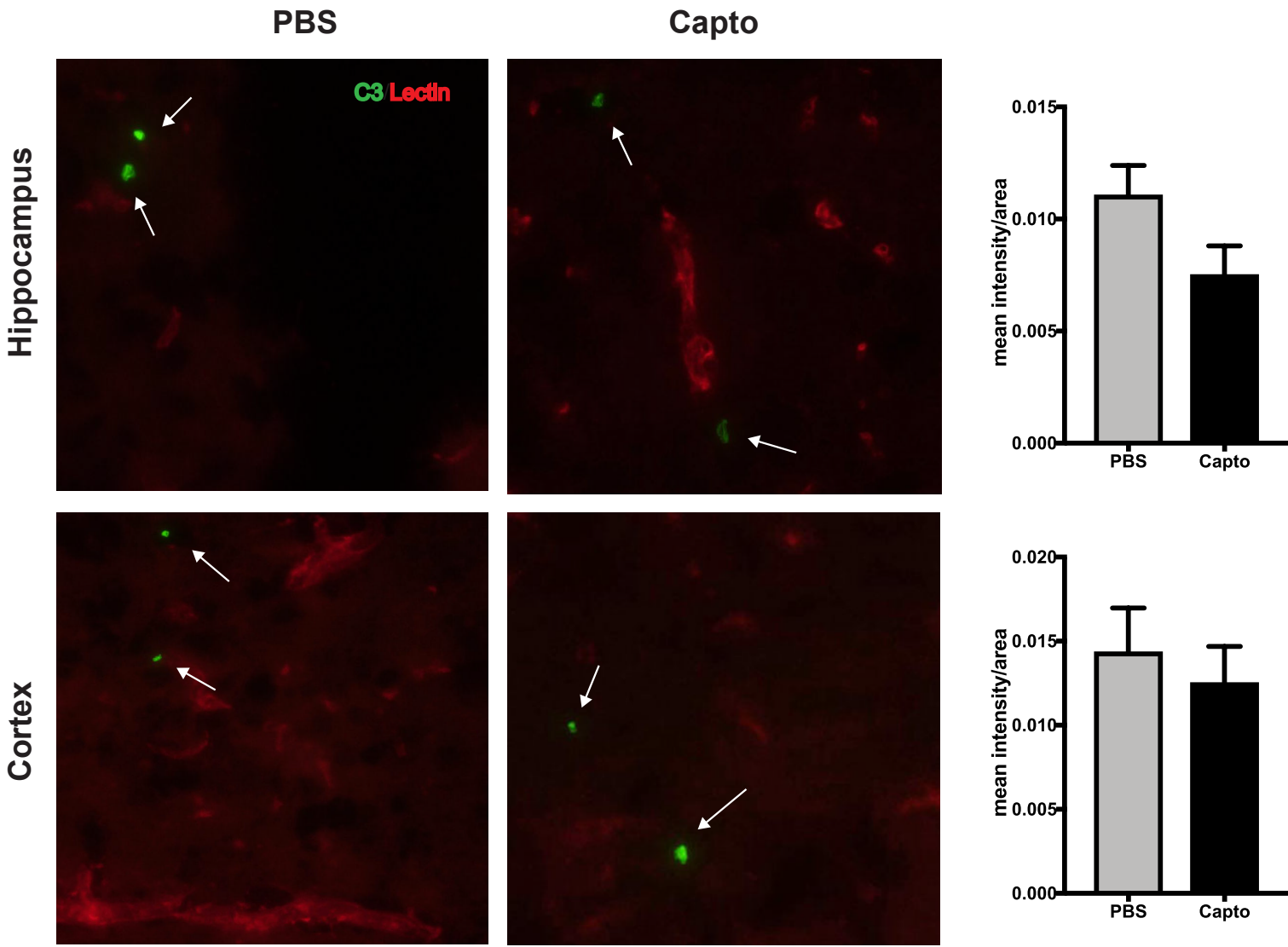

Supplement: FIGURE S3 — C3 deposition in MRL/lpr brain. Frozen brains from captopril-treated and control MRL/lpr mice were sagitally sectioned, stained for C3 (Alexa 488) and tomato lectin (Texas red) for microvessels, and analyzed in the cortex and hippocampal regions. Representative images (400x magnification) are shown for C3 in the cortex and hippocampal regions (indicated by arrows) of untreated mice and captopril-treated mice. Staining intensity was quantified by ImageJ. No significant difference in C3 staining was found between the treated and untreated mice. [file Data_Sheet_3.PDF]
